# Molecular epidemiology analysis of symptomatic and asymptomatic norovirus infections in Chinese infants

**DOI:** 10.1186/s12985-023-02024-z

**Published:** 2023-04-04

**Authors:** Li-Na Chen, Si-Jie Wang, Song-Mei Wang, Xiao-Li Fu, Wen-Jing Zheng, Zhi-Yong Hao, Hai-Song Zhou, Xin-Jiang Zhang, Yu-Liang Zhao, Chao Qiu, Lorenz von Seidlein, Tian-Yi Qiu, Xuan-Yi Wang

**Affiliations:** 1 Key Laboratory of Medical Molecular Virology of MoE & MoH and Institutes of Biomedical Sciences, Fudan University, 138 Yi Xue Yuan Rd., Shanghai, 200032 People’s Republic of China; 2 Laboratory of Molecular Biology, Training Center of Medical Experiments, School of Basic Medical Sciences, Fudan University, Shanghai, 200032 People’s Republic of China; 3 Zhengding County Center for Disease Control and Prevention, Zhengding, 050800 People’s Republic of China; 4 Hebei Province Center for Disease Control and Prevention, Shijiazhuang, 050021 People’s Republic of China; 5 Mahidol-Oxford Tropical Medicine Research Unit, Faculty of Tropical Medicine, Mahidol University, Bangkok, 73170 Thailand; 6 Institute of Clinical Science, ZhongShan Hospital, Fudan University, 180 Feng Ling Road, Shanghai, People’s Republic of China; 7 Shanghai Institute of Infectious Disease and Biosecurity, Shanghai, 200032 People’s Republic of China; 8 Children’s Hospital, Fudan University, Shanghai, 200032 People’s Republic of China

**Keywords:** Norovirus, Acute gastroenteritis, Birth cohort, Mutation, Recombination

## Abstract

**Background:**

Norovirus is a leading cause of acute gastroenteritis among children. Previous studies based on symptomatic infections indicated that mutations, rather than recombination drove the evolution of the norovirus ORF2. These characteristics were found in hospital-based symptomatic infections, whereas, asymptomatic infections are frequent and contribute significantly to transmission.

**Methods:**

We conducted the first norovirus molecular epidemiology analysis covering both symptomatic and asymptomatic infections derived from a birth cohort study in the northern China.

**Results:**

During the study, 14 symptomatic and 20 asymptomatic norovirus infections were detected in 32 infants. Out of the 14 strains that caused symptomatic infections, 12 strains were identified as GII.3[P12], and others were GII.4[P31]. Conversely, 17 asymptomatic infections were caused by GII.4[P31], two by GII.2[P16], and one by GII.4[P16]. Regardless of symptomatic and asymptomatic infections, the mutations were detected frequently in the ORF2 region, and almost all recombination were identified in the RdRp-ORF2 region. The majority of the mutations were located around the predefined epitope regions of P2 subdomain indicating a potential for immune evasion.

**Conclusion:**

The role of symptomatic as well as asymptomatic infections in the evolution of norovirus needs to be evaluated continuously.

**Supplementary Information:**

The online version contains supplementary material available at 10.1186/s12985-023-02024-z.

## Background

Norovirus a the nonenveloped, single-stranded positive-sense RNA virus belonging to the Caliciviridae family [1], is a leading cause for acute gastroenteritis (AGE) in human hosts worldwide, especially among children and the elderly [2–4]. The whole genome of norovirus is 7.7 kb, which can be organized into three open reading frames (ORF 1–3) [5]. Of these, ORF2 encodes the major capsid protein (VP1). X-ray crystallography of VP1 showed two structural domains, including the N-terminal shell (S) domain and the C-terminal protrusion (P) domain [6]. The P domain is further divided into the P1 subdomain and the variable P2 subdomain which contains putative neutralization sites and interacts with histo-blood group antigens (HBGAs) [7].

Norovirus continues to evolve in humans through both mutation and recombination, in particular, recombination appears to be a major force driving virus evolution [8]. Novel norovirus strains have emerged, and some classified into new tentative genogroups and genotypes [9–11]. In the past years, we also had studied the evolution of norovirus open reading frame 2 (ORF2), utilizing strains derived from population-based surveillance, and detected far more mutations than recombination events in RNA-dependent RNA polymerase (RdRp) region of ORF1 [12]. These evolutionary characteristics of norovirus are mostly based on symptomatic infections. Yet the fecal excretion of norovirus infection is common in asymptomatic individuals, especially in children [13]. Asymptomatic infections, constitute a significant reservoir for infection in the community, and may act as a source of endemic and epidemic disease [14, 15]. Our study characterized norovirus strains in samples collected weekly from symptomatic and asymptomatic infants participating in a birth cohort study. A phylogenetic analysis sequence encoding the norovirus RdRp and ORF2 was performed to classify the strains co-circulating during the surveillance period. The mutation and recombination patterns of norovirus strains were explored by recombination analysis.

## Materials and methods

### Study population and study design

To understand the transmission dynamics of norovirus in Chinese infants, a birth cohort study was conducted in 11 villages of Zhengding County, Hebei Province, China, which is located 270 km south of Beijing. Considering the potential interference of passively transferred maternal antibodies [16], infants aged between 3-months (90-days) and 4-months (120-days) were recruited. Exclusion criteria included serious medical conditions such as any neonatal disorder, kidney, liver, lung and/or heart disease, congenital disorders, clinically diagnosed enteropathy, and/or birth weight < 1.5 kg. Recruited infants were followed weekly for occurrence of AGE by trained doctors working in the village clinics. Stool specimens were collected from each infant once every week, and tested for norovirus using RT-PCR, regardless of the clinical manifestations of AGE. An AGE episode was defined as 3 or more loose bowel movements and/or 2 or more episodes of vomiting during a 24-h period [17]. Recovery of AGE was defined as the absence of loose stools and/or vomiting for 3 consecutive days. A symptomatic norovirus infection was defined as norovirus test-positive by RT-PCR in the stool samples collected from infants with AGE within 7 days after the onset, and an asymptomatic infection was defined correspondingly as norovirus test-positive by RT-PCR in the stool samples collected from infants without AGE [18].

This study was approved by the Institutional Review Board (IRB) of the Institutes of Biomedical Sciences, Fudan University. Written inform consent was obtained from a parent/guardian of each child included in the study. All CRFs were double entered into a custom-made data entry program (EpiData program, version 3.1) [19], the data management program included error and consistency checks.

### Norovirus RNA extraction and genotyping

Viral RNA was extracted from 10% of the stool supernatant using TianLong Stool DNA/RNA Extraction Kit (TianLong Science & Technology, China), as described previously [20]. The G (capsid type) and P (polymerase type) typing were carried out by amplifying parts of the ORF2 and ORF1 sequence, respectively. PCR assays was carried out using Qiagen One-Step RT-PCR kit (Qiagen, USA), with primer-pairs MON432/G1SKR for norovirus GI, or MON431/G2SKR for norovirus GII, generating 579-bp and 570-bp PCR products [21]. The assay was carried out in a 25 μl reaction mixture, containing 12.5 μl One-Step RT-PCR 2X Master Mix, 3 μl RNA template, 1.5 μl of 10 μM forward and reverse primers, and sterile, de-ionized water were mixed into a final volume of 25 μl. The cycling conditions included 30 min of reverse transcription at 42 °C, and then 15 min of initial denaturation at 95 °C. PCR amplification was performed for 40 cycles (95 °C, 50 °C, and 72 °C for 1 min each), and finally, reactions were completed with 10 min elongation at 72 °C followed by cooling to 4 °C. PCR products were analyzed on a 1 × TAE 2% agarose gel and visualized with Gel-Red staining. Dideoxy sanger sequencing of the products was performed by Tsingke (TsingkeBiotech, China). Genotypes were determined using the online Norovirus Genotyping Tool (https://www.rivm.nl/mpf/typingtool/norovirus/). Once the genotype was assigned, PCR assays were carried out using Qiagen One-Step RT-PCR kit (Qiagen, USA) with genotype-specific primers to amplify and sequence the full-length of ORF2 (Additional file 1: Table S1).

### Phylogenetic analysis

Phylogenetic trees were constructed using the neighbor-joining (NJ) method for each of the norovirus polymerase and capsid genes [22]. Multiple sequence alignments for phylogenetic analysis were performed with the Molecular Evolutionary Genetic Analysis software (MEGA, version 7.0.21) [23]. The best substitution model was selected based on the corrected Akaike’s information criterion (AICc) values [24]. Kimura-2 parameter models were selected, and gamma distributions were used to calculate genetic distance. Reliability analyses were performed using a bootstrap method. Sampling was repeated 1000 times, with less than 70% considered meaningless [25]. The sequences of norovirus strains used as reference were downloaded from GenBank database. The sequence of strains isolated from our study were available in the GenBank (GenBank accession nos. OQ451905–OQ451938).

### Nucleotide variation and protein mutation analysis

We evaluate the mutation and recombination events of the norovirus strains isolated in our study. The mutation was evaluated through Shannon entropy, which could represent the diversity of the mutation on individual site. In this study, the Shannon entropy was calculated by Shannon Entropy-One, which could apply phylogeny into Shannon entropy as a measure of variation in nucleotide sequence alignments (https://www.hiv.lanl.gov/content/sequence/ENTROPY/entropy_one.html) [26]. Entropy-one calculates the entropy at each position in the input sequence and can be used as a measure of the relative variation in different positions or regions of an aligned nucleotide sequence [27]. Entropy values for each position were plotted in GraphPad Prism v8 (San Diego, USA).

The ORF2 protein sequences of GII.4[P31] strains were aligned using MEGA and analyzed using AliView viewer and editing tool (version 1.28) [28]. Linear, discontinuous, and non-peptidic norovirus GII.4 specific T and B cell receptor epitopes mapping in VP1 region were retrieved from the Immune Epitope Database (IEDB) [29]. Mutations were defined as amino acid changes presenting between two or more VP1 sequences of GII.4 strains. The effect of the resulting amino acid substitutions on the structure and function of capsid proteins was prognosticated using the Polymorphism Phenotyping v2 (PolyPhen-2) software [30]. The prediction is based on a number of sequences, phylogenetic, and structural features characterizing the substitution. For a given amino acid substitution in a protein, PolyPhen-2 extracts various sequence and structure-based features of the substitution site and feeds them to a probabilistic classifier [31].

For structure modelling, the online server of SWISS-MODEL was used to construct the 3D structure of the VP1 protein through protein homology modeling with defaulted template [32]. Then, the potential deleterious positions can be visualized and annotated on the.pbd format files through PyMOL (version 2.3.4) [33].

### Recombination analysis

The identification of potential parental sequences and the localization of putative recombination breakpoints of norovirus strains isolated from our study were determined by the Recombination Detection Program version 4 (RPD4, version 101) [34]. Seven recombination detection methods (Bootscan, Chimaera, Geneconv, MaxChi, RDP, SISCAN, and 3Seq) in RPD4 were used to predict recombination events [35]. Recombination events were accepted when potential recombination signals (p-value cutoff of 0.05) detected by at least three out of the seven detection methods [36].

## Results

### Study population and characteristics

**Fig. 1:**
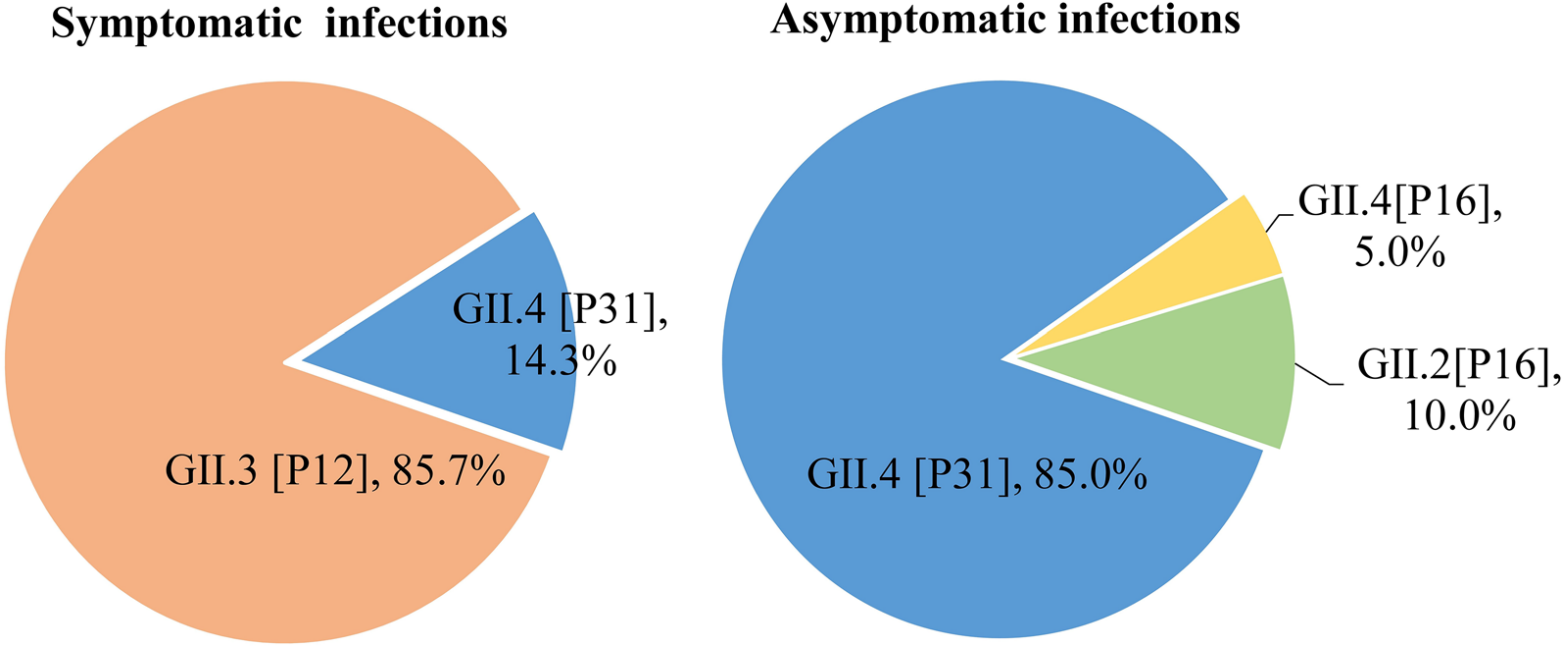
Distribution of norovirus genotypes in symptomatic and asymptomatic norovirus infections from a cohort study in China, from 2021–2022

As of the end of October, 100 infants were recruited from 11 villages. Between November 1, 2021 and March 1, 2022, which was the epidemic season, 1,600 fecal specimens were collected from the 100 infants. Of these, 14 symptomatic and 20 asymptomatic episodes of norovirus infections were detected in 32 infants, resulting in a cumulative infection rate of 34%. Compared with November (23.5%) and February (17.6%), norovirus infection was identified more frequently in December (26.5%) and January (32.4%). Re-infection was detected in 2 infants with different genotypes. One infant was identified as an asymptomatic GII.4[P16] infection, and subsequently experienced a symptomatic GII.3[P12] infection after 46 days. Another infant presented with diarrhea caused by GII.3[P12] infection, and then experienced an asymptomatic GII.2[P16] infection after 52 days. Out of 14 strains causing symptomatic infections, 12 strains were identified as GII.3[P12], and other strains were GII.4[P31]. Conversely, 17 asymptomatic infections were attributed to GII.4[P31], two as GII.2[P16], and one as GII.4[P16] (Fig. 1). The duration of viral shedding in symptomatic infections (1.43 ± 0.51 weeks) was slightly longer than that of asymptomatic infections (1.35 ± 0.49 weeks). This difference was not statistically significant.

### Phylogenetic analysis

**Fig. 2:**
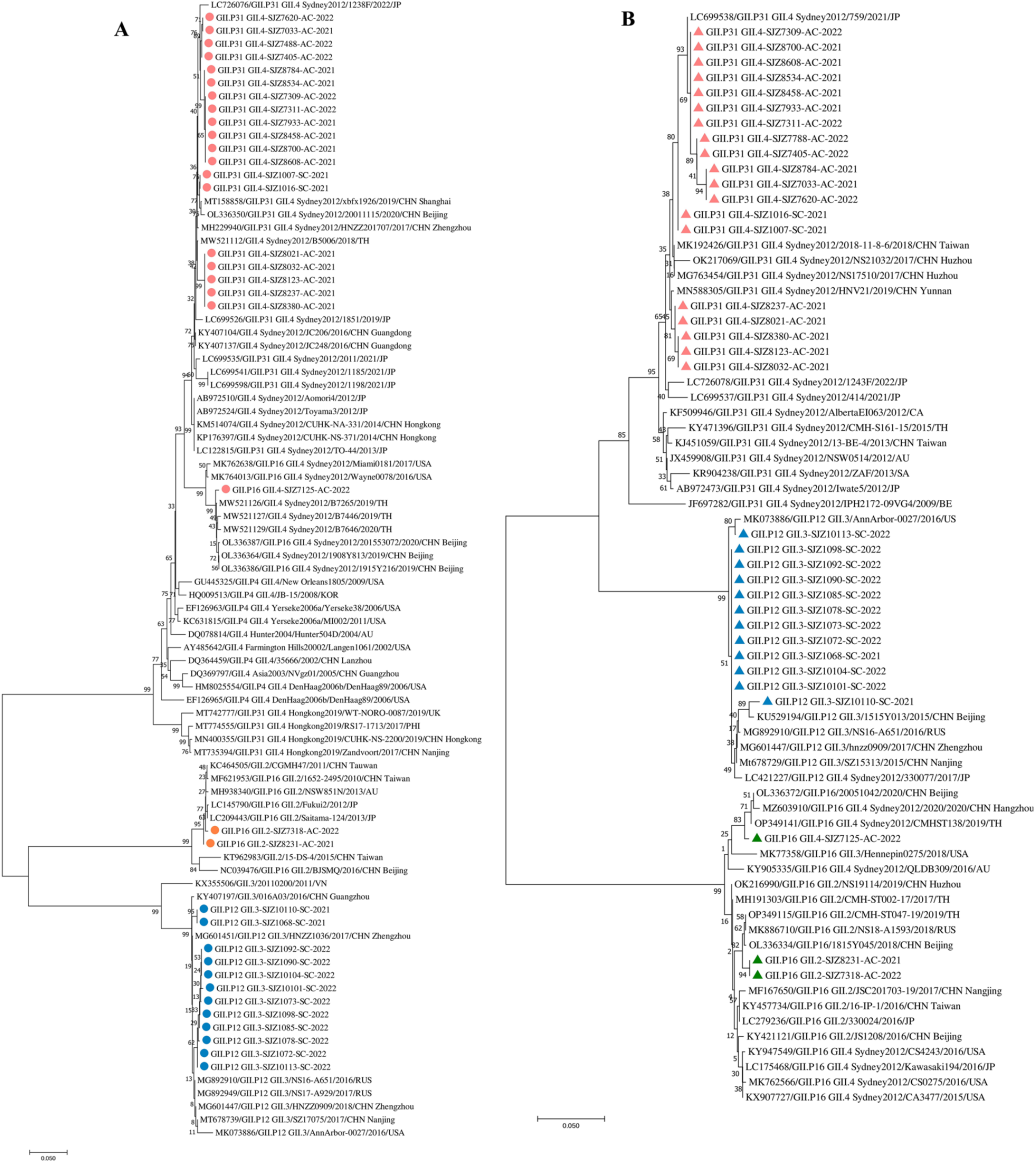
Phylogenetic analyses of norovirus isolated from symptomatic and asymptomatic infections in our cohort study during 2021–2022. **A** Phylogenetic tree based on the ORF2 nucleotide sequence **B** Phylogenetic tree based on the RdRp nucleotide sequence. Reference strains were downloaded from GenBank and indicated by GenBank accession number/strain/name/year/country (JP, Japan; CHN, China; USA, United States; TH, Thailand; KOR, Korea; AU, Australia; VN, Vietnam; RUS, Russia; CA, Canada). Strains obtained from our study are labeled as strain/sample name/source (AC, asymptomatic individuals; SC, symptomatic individuals)/year. GII.4 labeled as red circle; GII.2 labeled as orange circle; GII.3 labeled as blue circle; GII.P31 labeled as red triangle; GII.P12 labeled as blue triangle; GII.P16 labeled as green triangle. Neighbor-joining (NJ) phylogenetic tree was constructed with MEGA 7 software and bootstrap tests (1000 replicates), based on the Kimura-2 parameter model. Scale bar indicates distances between sequence pairs

To detect the relations between the previously worldwide circulating strains and the strains monitored through our cohort study, we constructed phylogenetic trees based on the partial sequences of ORF2 (1591nt) and RdRp (565nt) (Fig. 2). The majority of norovirus strains isolated from both symptomatic and asymptomatic individuals in our study belonged to norovirus GII.4 Sydney2012 (n = 20), including 19 GII.4[P31] and one GII.4[P16] (Fig. 2A). All norovirus strains with GII.4 Sydney2012 capsid type were clustered with the previously reported GII.4 strains from China, Japan, Philippine, Thailand, Korea and USA, creating a phylogenetically distinct monophyletic clade. The nucleotide sequence identities among the GII.4 Sydney2012 strains detected in this study range from 93.36 to 98.51%. The previously detected GII.4[P31] strains circulated from 2017 to 2022 in both northern and southern of China, including Beijing, Zhengzhou, Shanghai and Guangzhou, which were genetically close to the GII.4[P31] strains isolated in our study. Similar, the GII.4[P16] strain clustered with strains from China, Thailand and USA reported from 2016 to 2020. This finding suggests that this clade was imported between 2016 and 2019.

Besides GII.4 clades, we detected the second most-common genotype of GII.3 (n = 12) and the third most-common genotype of GII.2 (n = 2) in this study. The norovirus GII.3 strains were 97.3%-99.8% identical to each other and clustered together on the phylogenetic tree. The GII.3 strains detected in 2021 were clustered together with the HNZZ1036 GII.3 strain detected in Zhengzhou, China in 2017. The strains detected in 2022 were most closely related to the norovirus strains isolated in 2016 and 2017 in Russia. Two norovirus GII.2 strains were isolated from asymptomatic infected children in 2021 and 2022. The nucleotide sequence identity of the GII.2 strains isolated in our study was 99.4%. The strains were located in the same branch as norovirus GII.2 reference strains reported in 2010 and 2013 from China, Japan and Australia.

We detected three major clades of GII.P31, GII.P12 and GII.P16 based on the phylogenetic tree of the RdRp sequence (Fig. 2B). The majority of norovirus strains isolated in our study located in the GII.P31 clade (n = 19), which clustered with strains from China and Japan reported from 2017 to 2022. This branch also clustered with strains from Asia, Northern America and Europe isolated since 2009. Similarly, the second GII.3[P12] strains from SJZ were mostly located in one branch with high sequence identity (n = 12). Two other strains clustered with strains detected in the US and China, one GII.4[P16] strain and two highly similar GII.2[P16] strains.

### Nucleotide variation and protein mutation analysis

**Fig. 3:**
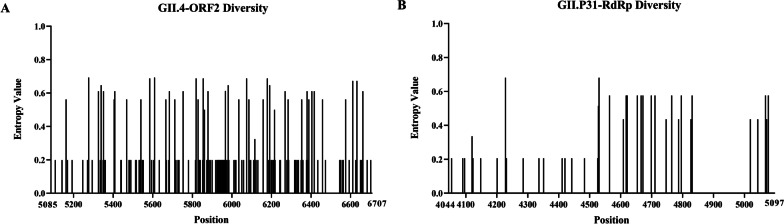
Site variability was calculated at nucleotide level using Shannon entropy for norovirus strains isolated from our study. Diversity nucleotide plots, were shown the difference site of the ORF2 (**A**) and RdRp (**B**) sequence. Sequence locus information is referenced to GII.4 Sydney 2012 genome (JX459908)

We evaluated the variation of norovirus strains isolated from our study at the nucleotide level. The Shannon entropy were illustrated in Fig. 3, including 20 strains of the GII.4 cluster based on ORF2 region (Fig. 3A) and 19 strains of the GII.P31 cluster based on RdRp region (Fig. 3B). The mean value of Shannon entropy spanning the whole GII.4 ORF2 region was 0.031 (range: 0–0.693), and the mean value across RdRp was 0.015 (range: 0–0.0681), suggesting that the polymerases proteins were relatively conserved. Results of other regions can be found in the Additional file 1: Fig. S1. As shown in the Fig. 3A, most variation were observed within ORF2, particularly in the 5900–6300 nucleotide sites, which located on the P2 subdomain.

To further determine whether nucleotide substitution led to amino acid variation, we analyzed the complete amino acid sequence of VP1 proteins of GII.4[P31] strains isolated from both symptomatic and asymptomatic infections in our study. The positions involving more than two mutated residues in different strains were considered significant mutation sites. Eight important mutation sites were detected in the VP1 protein. As shown in Table 1, 25.0% (2/8) amino acid changes were identified in the Shell domain of VP1 (aa site: 46–221), and 62.5% (5/8) were observed in the P2 subdomain (aa site: 276–417). The mutation site of V8A (1/8, 12.5%) was located in the N-terminal (aa site:1–45). No mutations were located in the P1 subdomain (aa site: 222–275 & 418–540).

**Table 1:** Mapping of immunogenic sites within VP1 region of different GII.4 strains in this study

Epitopes with mutations	Mutation Sites	Position of VP1 ^b^	Mutation Effect ^c^	PolyPhen-2 Score ^c^
*T cell restricted in immunodominant epitopes*				
SPSQVTMFPHIIVDVRQL_134–151_	I145V	Shell	Benign	0.356
VRNNFYHYNQSNDST_161–175_	S174P	Shell	Benign	0.010
GRNTHNVHL_410–418_	H414P ^a^	P2	Damaging	0.699
*B cell restricted in immunodominant epitopes*				
GDVTHITGSRNYTMNLASQNWSNY_288–311_	G295C	P2	Damaging	0.972
	S309N	P2	Benign	0.05
*Antibody epitopes*				
Epitope A_294–298, 368, 372–373_	N7373H	P2	Benign	0.237
Epitope D_393–397_	G393S	P2	Benign	0.143
Epitope E_407, 411–414_	H414P ^b^	P2	Damaging	0.699
*Other single amino acid substitutions*				
N/A	V8A	N	Damaging	0.571

CD4 + T restricted are in purple font, CD8 + T restricted are in blue font

Protein site information is referenced to GII.4 Sydney 2012 VP1 (AFV08795)

^a^Indicated residue targeted by both antibody and T cell receptor response

^b^N-terminal:1–45; Shell domain: 46–221; P1 subdomain: 222–275 & 418–540; P2 subdomain: 276–417

^c^Results from PolyPhen-2. Score goes from 0 to 1. Greater score indicates higher probability to impair the protein function

**Fig. 4:**
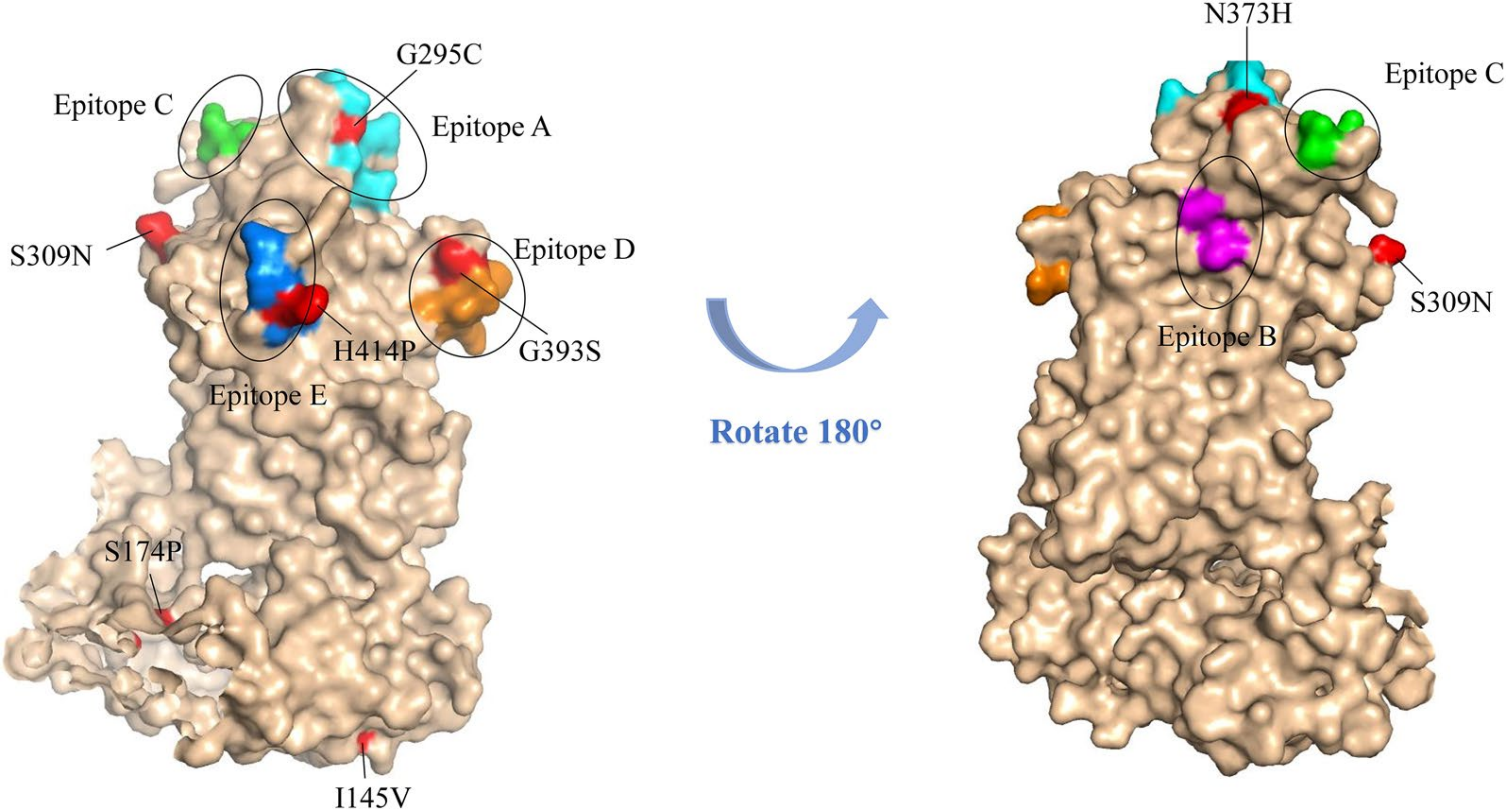
3D structure modeling of VP1 region of norovirus GII.4[P31] strain. Protein site information is referenced to GII.4 Sydney 2012 VP1 (AFV08795). Mutations identified in this study are shown in red. Epitopes A (294–298, 368, 372–373), B (333, 389), C (340, 376), D (393–395), and E (407, 411–414) are highlighted in cyan, magenta, green, orange, and blue, respectively. The 3D structures were generated with SWISS-MODEL and visualized using PyMOL (version 2.3.4)

In the shell domain, the S174P site was identified in the previously reported CD4 + T cell restricted epitopes _161_VRNNFYHYNQSNDST_175_ (IEDB ID: 985331), and the mutation site I145V was reported in the CD8 + T cell restricted epitope, _134_SPSQVTMFPHIIVDVRQL_151_ (IEDB ID: 561756). We detected the H414P mutation in both the CD8 + epitopes _410_GRNTHNVHL_418_ (IEDB ID: 984324) and antibody Epitopes E (407, 411–414) in the P2 subdomain. G295C and S309N, occurred in the B cell restrict epitopes of _288_GDVTHITGSRNYTMNLASQNWSNY_311_ (IEDB ID: 1334831). N373H was identified in the antibody Epitopes A (294–298, 368, 372–373) and G393S was identified in the antibody Epitopes D (393–397). Three mutations, V8A, G295C, H414P and were characterized in the PolyPhen-2 as damaging, and the rest were considered benign (Table 1). We mapped 5 previously determined epitope regions, labeled Epitope A to Epitope E on P2 subdomain (Fig. 4). By mapping the mutation sites on the P2 subdomain, we found most of the mutations were not directly located on the epitope regions, but close to them.

### Recombination analysis

**Fig. 5:**
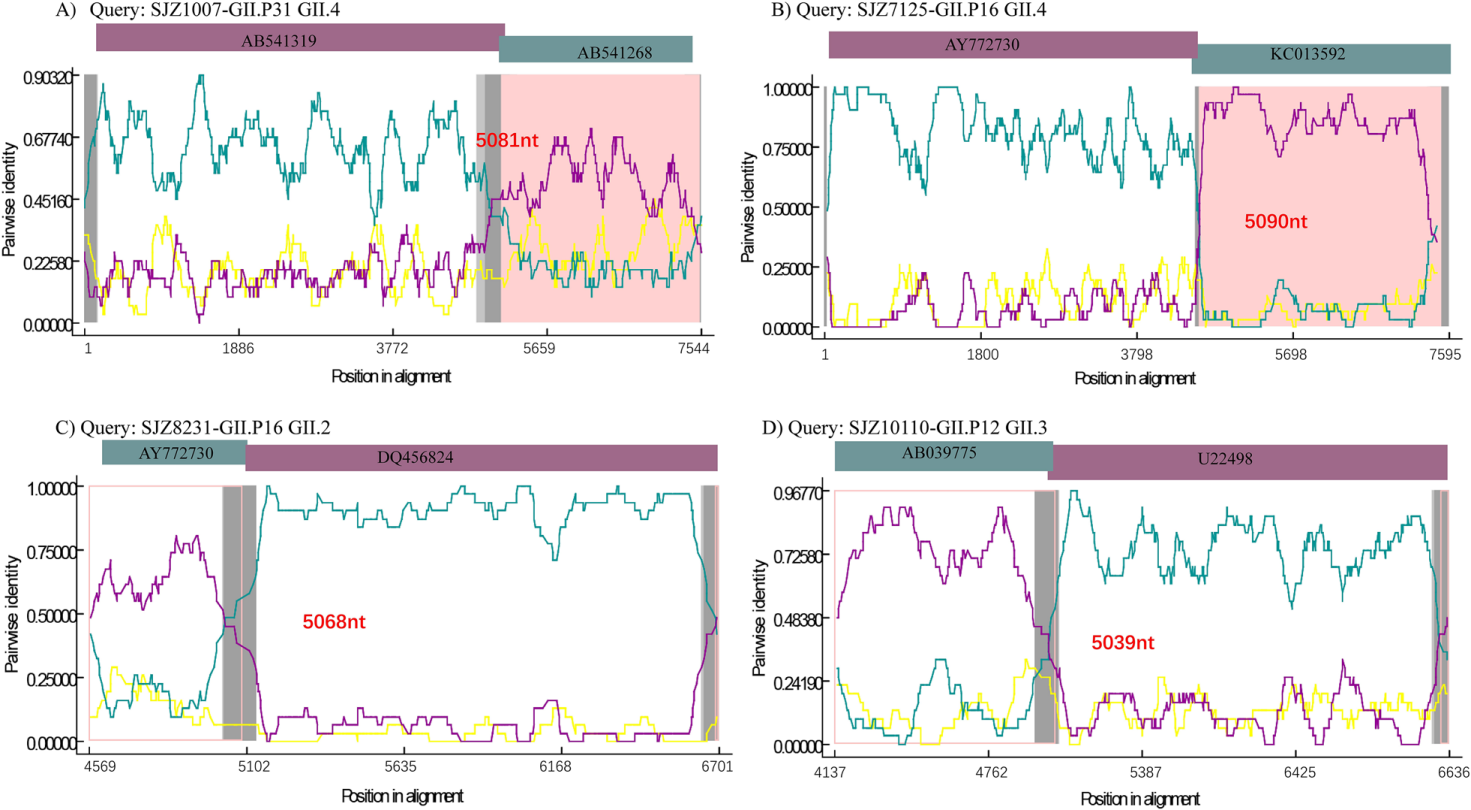
Genome recombination analysis of norovirus strains isolated from China in 2021–2022. The full length of the GII.4[P31] (SJZ1007) and GII.4[P16] (SJZ7125) sequence and partial of GII.2[P16] (SJZ8231) and GII.3[P12] (SJZ10110) were used for recombination analysis. RDP4 results show recombination breakpoints and relationships of representative strains to the parental strains. All plots were obtained using a 200 bp sliding window, 284 steps of 20 bp. Sequence locus information is referenced to GII.4 Sydney 2012 genome (JX459908). The horizontal line and vertical lines show nucleotide positions and nucleotide pair- wise identity. **A** Analysis of GII.4[P31] consensus sequence against Osaka_2007 strain (GenBank: AB541319, purple) and Apeldoorn_2008 strain (GenBank: AB541268, teal), predicted recombination position at 5081nt. **B** Analysis of GII.4[P16] consensus sequence against Berlin 2000 strain (GenBank: AY772730, purple) and US95_96 strain (GenBank: KC013592, teal), predicted recombination position at 5090nt. **C** Analysis of GII.2[P16] consensus sequence against Berlin 2000 strain (GenBank: AY772730, teal) and Tokyo 2004 strain (GenBank: DQ456824, purple), predicted recombination position at 5068nt. **D** Analysis of GII.3[P12] consensus sequence against Saitama 2002 strain (GenBank: AB039775, teal) and Mexican 1995 strain (GenBank: U22498, purple), predicted recombination position at 5039nt

Recombination is an alternative way for the norovirus to produce variants. We investigated the pattern of norovirus recombination by inclusion of both the norovirus strains identified from symptomatic and asymptomatic infants in cohort study. The results of recombination analysis can be found in Fig. 5 and the Additional file 1: Table S2. Two strains with full sequence length (Fig. 5A and B) and two strains with partial but complete RdRp/ORF2 sequence (Fig. 5C and 5D) were used for illustration. Results showed that the predicted recombination position is around 5020 to 5105, overlapped with the RdRp (4044–5097) and partial of ORF2 (5085–6707). Among all 34 isolated strains, the recombination position of 16 strains were located on RdRp, followed by 13 on the overlap region of RdRp and ORF2, and 5 on ORF2 (Additional file 1: Table S2). The results illustrated four possible recombinants GII.4[P31], GII.4[P16], GII.2[P16] and, GII.3[P12]. For GII.4[P31], the parental strains were Osaka_2007 (AB541319) for ORF1 and Apeldoorn_2008 (AB541268) for ORF2-ORF3, which are the common parental strains for other GII.4[P31] subtypes (Fig. 5A and Additional file 1: Table S2). The ORF1 parental strain for the GII.P16 strain was AY772730 strain from Germany in 2000, which has different recombination points from the GII.4[P16] strain of SJZ7125 (Recombination breakpoint site 5090) and the GII.2[P16] strain of SJZ8231 (Recombination breakpoint site 5068) (Fig. 5B). The ORF2-ORF3 parental strain for the GII.4[P16] strain of SJZ7125 was predicted as US95_96 (KC013592), which suggested a different origin than the GII.2[P16] strain of SJZ8231 with ORF2 origin from Japan in 2004 (DQ456824) (Fig. 5C). We found that all 12 GII.3[P12] strains originated from parental Norwalk-like strains isolate from Japan in 2000 (AB039775) and Mexican in 1995 (U22498) (Fig. 5D).

## Discussion

Norovirus is a leading cause of AGE, especially among children in China [37]. For a comprehensive understanding of virus evolution and transmission dynamics in symptomatic and asymptomatic infections, a birth cohort study is desirable [3]. Our previous study estimated that norovirus infection led to an annual incidence rate of 6.0, 15.6 and 5.5 per 100 persons per year in the three age groups a) all ages, b) less than 5 years and c) older than 60 years respectively [38]. We have studied the evolution of the major capsid protein (VP1) encoded by the ORF2 based on sequences isolated from population-based diarrhea surveillance in Zhengding county spanning between 2001 and 2019, and concluded that antigenic variation was the major mechanism for the emergence of novel VP1s (not including RdRp region), rather than recombination [12]. However, all of these findings were derived from symptomatic infections caused by norovirus. This study is the first birth cohort study to explore the transmission dynamics of norovirus in Chinese infants. More asymptomatic infections (20%) than symptomatic infections (14%), were detected, which suggested that the asymptomatic infections played an important role in the persistence of transmission, and thus, should be accounted when exploring the evolution of norovirus [39, 40]. Several factors might be involved in the mechanism for the high occurrence of asymptomatic infection in the community. Firstly, the infants recruited in our study were around 6 months of age when they experienced the epidemic season. Some studies have demonstrated that the maternal antibody decay rapidly in the first months of life, although the measurable maternal antibodies were expected to have waned at 6 months [41–44], and thus could still provide a certain level of protection, and eventually attenuated the severity of infections [45, 46]. Secondly, the study site is located in rural areas, where infants are generally breastfed up to 12 months of age. It was reported that norovirus-specific immunoglobulin A in breast milk might protect against norovirus associated diarrhea but not norovirus infection [47]. Similar to some published studies [48, 49], symptomatic infections were not associated significantly with duration of viral shedding.

In this study, 14 strains that caused symptomatic infections were identified as GII.3[P12] and GII.4[P31], while 20 strains that caused asymptomatic infections were involving GII.4[P31], GII.2[P16] and GII.4[P16]. Among them, GII.4 Sydney2012 strains were detected in both AGE patients and asymptomatic carriers, which is in agreement with previous studies [50–52]. Since first detected in USA in 2012, GII.4 Sydney[P31] strains have been the predominated strain resulting in global pandemics [53]. The phylogenetic tree suggested that GII.4[P31] strains isolated from our study have a close genetic distance with previously circulating strains in Shanghai, Beijing, Zhengzhou and Guangzhou in 2016–2020. This finding proves that GII.4[P31] strains have circulated in the mainland China before 2016 [54, 55]. The GII.4[P16] strain in our study was similar to the viruses circulating in Beijing in 2019–2020, and strains detected in Thailand in 2019–2020. It has been reported that the GII.4 Sydney[P16] strain emerged in 2015 and was soon replaced by the GII.4 Sydney[P31] strain as the primary cause of outbreaks in the United States [56]. Despite the fact that GII.4 Sydney[P16] strains were rarely detected in our study, it remains necessary to monitor the prevalence of this strain. GII.3[P12] strains in our study clustered with strains from China, Japan, Russia and the United States in 2015–2018, and GII.2[P16] strains were clustered with China, Thailand and Japan in 2011–2013, suggesting that the strains were spread across the globe. We found that the GII.3[P12] strains were only detected in symptomatic infections, and GII.2[P16] strains were only detected in asymptomatic infections. This result may provide new perspective for the investigation of symptomatic and asymptomatic infections, but the results in this study are not sufficient to provide that the infection patterns are specifically distinguishable for those two genotypes. In the future, with the increasing accumulation of molecular epidemiological data, it may be possible for us to verify whether symptomatic and asymptomatic infections are related to the specific genotypes of norovirus.

The emergence and spread of novel norovirus strains is associated with point mutations in ORF1 and ORF2 region, and recombination events that produce chimeric viruses [57]. Although all norovirus utilized both mechanisms, different genotypes may preferentially emerge and persist in populations. Our study found that the mutation frequency of ORF2 region was higher than RdRp region of the ORF1, especially in the P2 subdomain. The amino acid mutations of the VP1 protein identified in our study (amino acid positions 309, 373, 414) corresponded to the same characteristics identified by another Chinese group, and the remaining amino acid mutation sites were located at or near the amino acids identified by this group [55]. Most of these mutations are located at or near the epitope region of the P2 sub-domain. It has been reported that residue changes of the epitope region are likely to enable the norovirus to escape the pressure of population immunity and cause global epidemic [58, 59]. The predicted recombination breakpoints in norovirus strains identified from symptomatic and asymptomatic infants in our study around 5020 to 5105nt, overlapped with the RdRp (4044–5097) and partially overlapped with ORF2 (5085–6707). This is consistent with previously reported strains isolated during gastroenteritis outbreaks [60]. RdRp of norovirus is a key enzyme responsible for viral transcription and replication and was suggested to be a driving factor in norovirus recombination [61]. Norovirus recombination typically occurs at the RdRp-ORF2 junction [62, 63], which is also the transcription initiation site for viral sub-genomic RNA. It has been shown that exchanges in RdRp region caused by genomic recombination result in an increased mutation rate through acquisition of an RdRp with lower fidelity and/or increased replicative ability, improving viral fitness under certain selective pressures and hence drive virus evolution [64]. Therefore, recombination may be one of the reason for the diversity of norovirus, which not only causing the genomic exchange of RdRp region, but also increasing the mutation rate in other regions such as VP1 [65]. Meanwhile, mutations in regions other than RdRp, may be caused by antigenic drift or shift that result in point mutations to escape immune response [57]. Together, the recombination and the point mutation may lead to the generation of new immune escape strains. Thus, large molecular epidemiological cohort studies are required to further verify the causes of norovirus diversity.

## Conclusions

In conclusion, during the study period, more episodes of asymptomatic infection were detected in comparison with that of symptomatic infection. Genetic diversity between isolated strains of symptomatic and asymptomatic infection was not observed. The predominant circulating strains were GII.4, followed by GII.3 and GII.2, which origin possibly for recombination include GII.4[P31], GII.4[P16], GII.2[P16], and GII.3[P12] circulating in China, and other Asian countries in recent years. The role of asymptomatic infection in the evolution and transmission of noroviruses needs to be evaluated continuously.

## Supplementary Information

**Additional file 1: Table S1.** Genotype-specific primers were used to amplify and sequence the full-length of ORF2. **Table S2.** Analysis of predicted recombinant breakpoints of norovirus strains isolated from a cohort study, between 2021 and 2022. **Fig. S1.** Site variability was calculated at nucleotide level using Shannon entropy for norovirus strains isolated from our study. Diversity nucleotide plots, were shown the difference sites of the ORF2 and RdRp of norovirus GII.3[P12] and GII.2[P16] sequences. Sequence locus information is referenced to GII.4 Sydney2012 genome (JX459908)

## Data Availability

All data were collected from publicly available literatures, and all data generated or analyzed during this study are included in this published article and its additional files.

## Abbreviations

AGE: Acute gastroenteritis
RT-PCR: Reverse transcription-polymerase chain reaction
RdRp: RNA-dependent RNA polymerase
