# Supplementary material for: Molecular epidemiology analysis of symptomatic and asymptomatic norovirus infections in Chinese infants
Source: Virol J. 2023 Apr 4;20:60. doi: 10.1186/s12985-023-02024-z (PMC10074819; doi:10.1186/s12985-023-02024-z)
Supplement: Supplementary file 1 — Additional file 1: Table S1. Genotype-specific primers were used to amplify and sequence the full-length of ORF2. Table S2. Analysis of predicted recombinant breakpoints of norovirus strains isolated from a cohort study, between 2021 and 2022. Fig. S1. Site variability was calculated at nucleotide level using Shannon entropy for norovirus strains isolated from our study. Diversity nucleotide plots, were shown the difference sites of the ORF2 and RdRp of norovirus GII.3[P12] and GII.2[P16] sequences. Sequence locus information is referenced to GII.4 Sydney2012 genome (JX459908) [file 12985_2023_2024_MOESM1_ESM.docx]

**Supplementary Table S1.** Genotype-specific primers were used to amplify and sequence the full-length of ORF2

| Genotype | Polarity | Sequence (5’-3’) |
| --- | --- | --- |
| GII.2[P16] | (+) | GAAGGTGGGATGGACTTTTAC |
|  | (-) | TCTGCCCTCTGATTTATTGCAT |
| GII.3[P12] | (+) | TTGCAGAGTTGAAGGAAGGTGGCATGGATTT |
| GII.4[P31] | (+) | TTGCAGAGTTGAAGGAAGGTGGCATGGATT |
| GII.4[P16] | (+) | GAATGAAGATGGCGTCGAGTGA |
| Tx30SXN | (-) | GACTAGTTCTAGATCGCGAGCGGCCGCCCT(30) |

**
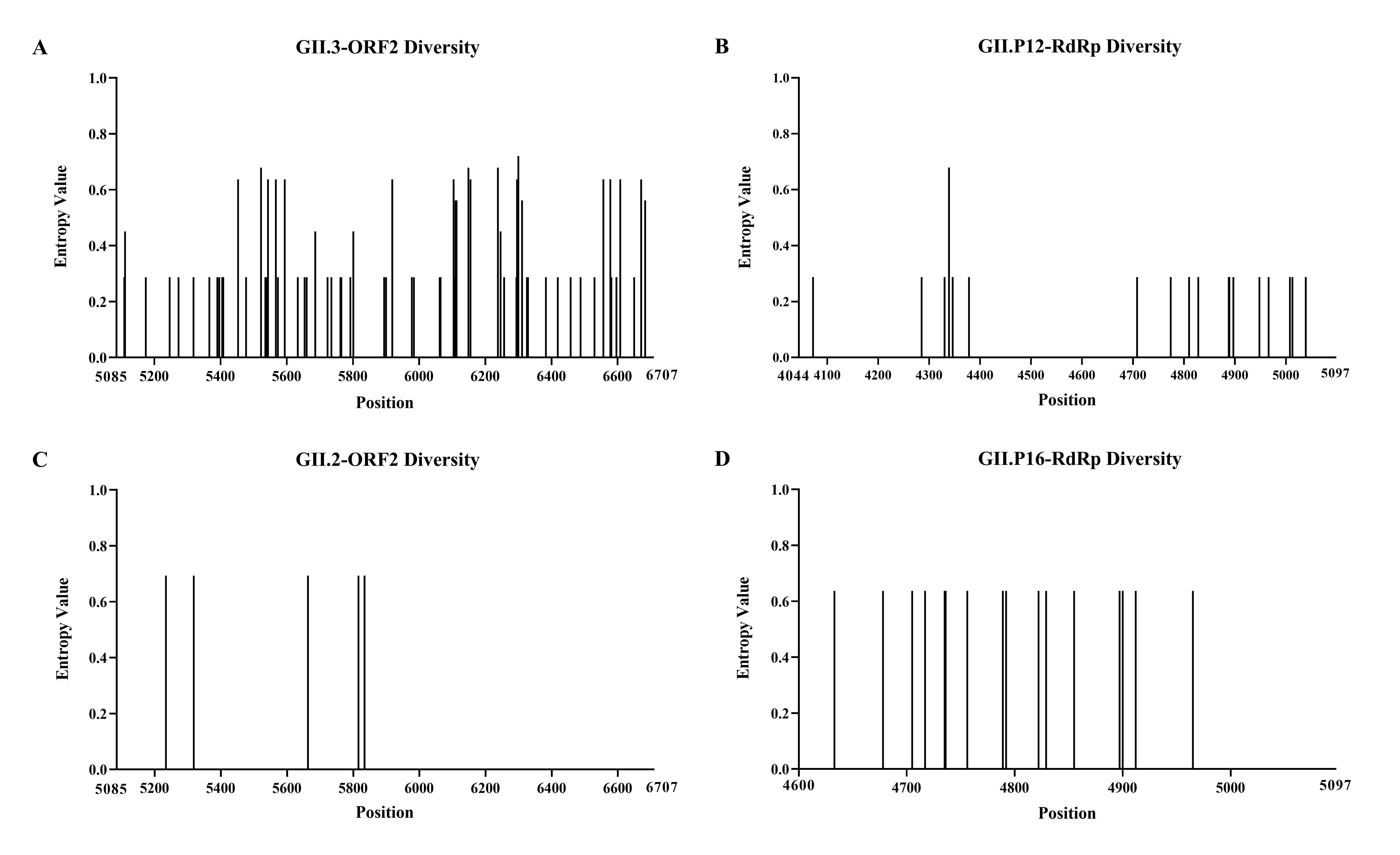
**

**Supplementary Figure S1**. Site variability was calculated at nucleotide level using Shannon entropy for norovirus strains isolated from our study. Diversity nucleotide plots, were shown the difference sites of the ORF2 and RdRp of norovirus GII.3[P12] and GII.2[P16] sequences. Sequence locus information is referenced to GII.4 Sydney 2012 genome (JX459908).

**Supplementary Table S2.** Analysis of predicted recombinant breakpoints of norovirus strains isolated from a cohort study, between 2021 to 2022.

| Sample code | Source^a^ | Length (nt) | Position^a^ | Norovirus genotypes | | Reference strains used for RDP4 analysis  (Accession no.) | | Predicted recombination nt positions^b^ | Breakpoint on ORF | p-value (RDP) |
| --- | --- | --- | --- | --- | --- | --- | --- | --- | --- | --- |
|  |  |  |  | ORF1 (RdRp) | ORF2 (VP1) | RdRp genotype | VP1 genotype |  |  |  |
| SJZ1007 | SC | 7548 | 5-7552 | GII.P31 | GII.4 | GII.4[P31] (AB541319) | GII.4[P4] (AB541268) | 5081 | RdRp | 1.94 x 10^-7^ |
| SJZ1016 | SC | 2668 | 4004-6667 | GII.P31 | GII.4 | GII.4[P31] (AB541319) | GII.4[P4] (AB541268) | 5081 | RdRp | 1.20 x 10^-3^ |
| SJZ7620 | AC | 2664 | 4044-6711 | GII.P31 | GII.4 | GII.4[P31] (AB541319) | GII.4[P4] (AB541268) | 5090 | RdRp/ORF2 | 1.36 x 10^-3^ |
| SJZ7033 | AC | 2664 | 4044-6711 | GII.P31 | GII.4 | GII.4[P31] (AB541319) | GII.4[P4] (AB541268) | 5090 | RdRp/ORF2 | 5.96 x 10^-4^ |
| SJZ7488 | AC | 2664 | 4044-6711 | GII.P31 | GII.4 | GII.4[P31] (AB541319) | GII.4[P4] (AB541268) | 5090 | RdRp/ORF2 | 1.52 x 10^-3^ |
| SJZ7405 | AC | 2664 | 4044-6711 | GII.P31 | GII.4 | GII.4[P31] (AB541319) | GII.4[P4] (AB541268) | 5090 | RdRp/ORF2 | 1.74 x 10^-3^ |
| SJZ8784 | AC | 2664 | 4044-6711 | GII.P31 | GII.4 | GII.4[P31] (AB541319) | GII.4[P4] (AB541268) | 5090 | RdRp/ORF2 | 1.18 x 10^-3^ |
| SJZ8534 | AC | 2664 | 4044-6711 | GII.P31 | GII.4 | GII.4[P31] (AB541319) | GII.4[P4] (AB541268) | 5090 | RdRp/ORF2 | 5.63 x 10^-4^ |
| SJZ7309 | AC | 2664 | 4044-6711 | GII.P31 | GII.4 | GII.4[P31] (AB541319) | GII.4[P4] (AB541268) | 5090 | RdRp/ORF2 | 8.17 x 10^-4^ |
| SJZ7311 | AC | 2664 | 4044-6711 | GII.P31 | GII.4 | GII.4[P31] (AB541319) | GII.4[P4] (AB541268) | 5090 | RdRp/ORF2 | 1.07 x 10^-3^ |
| SJZ7933 | AC | 2664 | 4044-6711 | GII.P31 | GII.4 | GII.4[P31] (AB541319) | GII.4[P4] (AB541268) | 5090 | RdRp/ORF2 | 1.31x 10^-4^ |
| SJZ8458 | AC | 2664 | 4044-6711 | GII.P31 | GII.4 | GII.4[P31] (AB541319) | GII.4[P4] (AB541268) | 5090 | RdRp/ORF2 | 2.79x 10^-4^ |
| SJZ8700 | AC | 2664 | 4044-6711 | GII.P31 | GII.4 | GII.4[P31] (AB541319) | GII.4[P4] (AB541268) | 5090 | RdRp/ORF2 | 1.07 x 10^-3^ |
| SJZ8608 | AC | 2664 | 4044-6711 | GII.P31 | GII.4 | GII.4[P31] (AB541319) | GII.4[P4] (AB541268) | 5090 | RdRp/ORF2 | 4.04 x 10^-4^ |
| SJZ8021 | AC | 2642 | 4044-6685 | GII.P31 | GII.4 | GII.4[P31] (AB541319) | GII.4[P4] (AB541268) | 5105 | ORF2 | 3.44 x 10^-5^ |
| SJZ8032 | AC | 2642 | 4044-6685 | GII.P31 | GII.4 | GII.4[P31] (AB541319) | GII.4[P4] (AB541268) | 5105 | ORF2 | 2.24 x 10^-5^ |
| SJZ8123 | AC | 2642 | 4044-6685 | GII.P31 | GII.4 | GII.4[P31] (AB541319) | GII.4[P4] (AB541268) | 5105 | ORF2 | 2.18 x 10^-5^ |
| SJZ8237 | AC | 2642 | 4044-6685 | GII.P31 | GII.4 | GII.4[P31] (AB541319) | GII.4[P4] (AB541268) | 5105 | ORF2 | 2.40 x 10^-5^ |
| SJZ8380 | AC | 2642 | 4044-6685 | GII.P31 | GII.4 | GII.4[P31] (AB541319) | GII.4[P4] (AB541268) | 5105 | ORF2 | 2.24 x 10^-5^ |
| SJZ7125 | AC | 7590 | 5-7594 | GII.P16 | GII.4 | GII.16[P16] (AY772730) | GII.4[P4] (KC013592) | 5090 | RdRp/ORF2 | 8.39 x 10^-29^ |
| SJZ7318 | AC | 2134 | 4569-6702 | GII.P16 | GII.2 | GII.16[P16] (AY772730) | GII.2[P2] (DQ456824) | 5068 | RdRp | 2.66 x 10^-19^ |
| SJZ8231 | AC | 2137 | 4569-6705 | GII.P16 | GII.2 | GII.16[P16] (AY772730) | GII.2[P2] (DQ456824) | 5068 | RdRp | 4.38 x 10^-18^ |
| SJZ10110 | SC | 2499 | 4137-6635 | GII.P12 | GII.3 | GII.12[P12] (AB039775) | GII.3[P3] (U22498) | 5039 | RdRp | 6.04 x 10^-43^ |
| SJZ1068 | SC | 2499 | 4137-6635 | GII.P12 | GII.3 | GII.12[P12] (AB039775) | GII.3[P3] (U22498) | 5039 | RdRp | 1.69 x 10^-45^ |
| SJZ10101 | SC | 2499 | 4137-6635 | GII.P12 | GII.3 | GII.12[P12] (AB039775) | GII.3[P3] (U22498) | 5020 | RdRp | 6.04 x 10^-43^ |
| SJZ1098 | SC | 2498 | 4138-6635 | GII.P12 | GII.3 | GII.12[P12] (AB039775) | GII.3[P3] (U22498) | 5020 | RdRp | 2.30 x 10^-45^ |
| SJZ1092 | SC | 2498 | 4138-6635 | GII.P12 | GII.3 | GII.12[P12] (AB039775) | GII.3[P3] (U22498) | 5020 | RdRp | 3.91 x 10^-45^ |
| SJZ1090 | SC | 2495 | 4141-6635 | GII.P12 | GII.3 | GII.12[P12] (AB039775) | GII.3[P3] (U22498) | 5020 | RdRp | 1.36 x 10^-45^ |
| SJZ1072 | SC | 2495 | 4141-6635 | GII.P12 | GII.3 | GII.12[P12] (AB039775) | GII.3[P3] (U22498) | 5020 | RdRp | 1.32 x 10^-44^ |
| SJZ10104 | SC | 2494 | 4142-6635 | GII.P12 | GII.3 | GII.12[P12] (AB039775) | GII.3[P3] (U22498) | 5020 | RdRp | 1.09 x 10^-44^ |
| SJZ1073 | SC | 2494 | 4142-6635 | GII.P12 | GII.3 | GII.12[P12] (AB039775) | GII.3[P3] (U22498) | 5020 | RdRp | 3.89 x 10^-45^ |
| SJZ1085 | SC | 2494 | 4142-6635 | GII.P12 | GII.3 | GII.12[P12] (AB039775) | GII.3[P3] (U22498) | 5020 | RdRp | 8.62 x 10^-45^ |
| SJZ1078 | SC | 2494 | 4142-6635 | GII.P12 | GII.3 | GII.12[P12] (AB039775) | GII.3[P3] (U22498) | 5020 | RdRp | 4.72 x 10^-45^ |
| SJZ10113 | SC | 2492 | 4144-6635 | GII.P12 | GII.3 | GII.12[P12] (AB039775) | GII.3[P3] (U22498) | 5020 | RdRp | 4.93 x 10^-45^ |

^a^ Sources of norovirus strains isolated in our study. Abbreviation: AC, asymptomatic infection; SC, symptomatic infection.

^b^ Sequence locus information is referenced to GII.4 Sydney 2012 genome (JX459908).
